# Dealing with uncertainties in environmental burden of disease assessment

**DOI:** 10.1186/1476-069X-8-21

**Published:** 2009-04-28

**Authors:** Anne B Knol, Arthur C Petersen, Jeroen P van der Sluijs, Erik Lebret

**Affiliations:** 1 National Institute for Public Health and the Environment (RIVM), Bilthoven, the Netherlands; 2 Netherlands Environmental Assessment Agency (PBL), Bilthoven, the Netherlands; 3 Copernicus Institute for Sustainable Development and Innovation, Utrecht University, the Netherlands; 4 Centre d'Economie et d'Ethique pour l'Environnement et le Développement, Université de Versailles Saint-Quentin-en-Yvelines, France; 5 Institute for Risk Assessment Sciences (IRAS), Utrecht University, the Netherlands

## Abstract

Disability Adjusted Life Years (DALYs) combine the number of people affected by disease or mortality in a population and the duration and severity of their condition into one number. The environmental burden of disease is the number of DALYs that can be attributed to environmental factors. Environmental burden of disease estimates enable policy makers to evaluate, compare and prioritize dissimilar environmental health problems or interventions. These estimates often have various uncertainties and assumptions which are not always made explicit. Besides statistical uncertainty in input data and parameters – which is commonly addressed – a variety of other types of uncertainties may substantially influence the results of the assessment. We have reviewed how different types of uncertainties affect environmental burden of disease assessments, and we give suggestions as to how researchers could address these uncertainties. We propose the use of an uncertainty typology to identify and characterize uncertainties. Finally, we argue that uncertainties need to be identified, assessed, reported and interpreted in order for assessment results to adequately support decision making.

## Background

In environmental health research, focus has shifted from relatively simple to more complex issues. Empirical single agent – single effect studies have been supplemented by research on risks of complex environmental exposures in varying economic, cultural and political settings. Environmental health impact assessment has become a valuable tool for decision support. These types of assessments increasingly use so-called environmental burden of disease (eBoD) measures to express health impacts. The eBoD can be viewed as the gap – caused by environmental factors – between current health status and an alternative situation in which environmental exposures are reduced or eliminated. Burden of disease estimates enable comparison of divergent environmental health problems. This in turn enables policy makers to set priorities. However, scientists often have to make many assumptions when assessing the eBoD. Knowledge and data are often incomplete, and diverging perceptions exist about what the most important aspects of a problem are. Assessments are often highly interdisciplinary, complex and multifaceted, and the uncertainty about results can be significant [1]. This may affect decision making based on these assessments.

A 2005 comparison of 17 eBoD studies published between 1996 and 2005 (internal RIVM/MNP publication by Knol et al.) showed that there are significant differences between eBoD estimates that concern – at first sight – similar issues. Smith et al. [2], for example, estimate the fraction of the total global disease burden attributable to the environment to be 25–33%, whereas Melse and de Hollander [2,3] estimate this to be 7.5 to 11% (for OECD countries only: 2–5%). Such differences can sometimes not be fully explained by reading the assessment reports. Methods, assumptions and input data are often insufficiently explained, which hampers interpretation and comparability of results. Fox-Rushby and Hanson [4] show that 9 out of 16 papers on burden of disease published between 1993 and 2000 did not declare the underlying assumptions.

Even though it is never possible to reduce uncertainty to zero in these complex assessments, there is significant room for improvement in dealing with uncertainty [1]. Various eBoD studies have addressed the need for uncertainty and sensitivity analyses (for example [2,4-10]), but, as yet, these analyses are based primarily on statistical uncertainty of some parameters and input data. Other sources of uncertainty are often touched upon in the discussion sections of publications, but usually not in a systematic manner. However, many environmental health issues are not straightforward and uncertainties cannot be captured in simple confidence intervals [11,12]. Only if both scientists and policy makers realize the potential extent of uncertainties and the way they may affect the assessment results, can these assessments lead to truly informed policy making. In order to achieve this, a typology of different dimensions of uncertainty can help to structure, assess and potentially reduce uncertainties, and moreover to improve the dialogue about uncertainties between scientists and policy makers.

The present study explores the different types of uncertainty that may play a role in eBoD studies expressed in Disability Adjusted Life Years, structured using a typology. The impact that uncertainties can have on assessment results – and thereby on decision making – will be illustrated using examples from the existing eBoD literature. Some suggestions are given as to how to address and communicate uncertainties to policy makers. This paper aims to create awareness among environmental health impact assessors about the potential impact and importance of uncertainties, and to provide a practical approach and structure to deal with uncertainties in eBoD assessments.

### Disability Adjusted Life Years

An increasingly popular metric to express the environmental burden of disease is the DALY (Disability Adjusted Life Years). DALYs indicate the potential number of healthy life years lost in a population, i.e. burden of disease. Not only life years lost due to premature mortality, but also years spent with reduced quality of life due to diseases are included. For diseases, severity weights (also referred to as disability weights) are used to quantify the reduced quality of life. They are developed by expert panels and range from 0 for complete health to 1 for death. Diseases with a severity weight ranging from 0.05 to 0.1 include for example low back pain, uncomplicated diabetes, or mild angina. Examples of more severe diseases with weights ranging from 0.65 to 0.8 include cancer, severe depression, and brain injury. These specific weights have been derived by Stouthardt et al. [13] and it should be recognized that other authorities might assign different weights to these effects.

Additionally, DALY calculations can include age weights and discounting factors. Age weighting involves valuing life years lost at a certain age more than life years lost at other ages. Discount factors are used to value present years of life saved more than future years. The usual annual discount rate is 3%, implying that a year of healthy life gained in 10 years time is valued at 26 percent less than one gained now. The use of age weights and discount factors has been discussed – and heavily debated – elsewhere (for example [14,15]).

Burden of disease calculations using DALYs were first published in the World Development Report [16]. Subsequently, Murray and Lopez [17] used DALYs in their extensive Global Burden of Disease project in order to introduce morbidity into the predominantly mortality-based health discussions. Since then, the World Health Organization (WHO) has endorsed the DALY approach, and it has been used in various studies on global, national and regional levels [3,18-26]. Burden of disease calculations are now increasingly being asked for in order to develop, evaluate and prioritize health-related policy measures. As well as DALYs, various other summary measures exist to express population health or disease states, such as QALYs (Quality Adjusted Life Years), HALYs (Health Adjusted Life Years), DALEs (Disability Adjusted Life Expectancy), HALEs (Health Adjusted Life Expectancy), and various monetary valuation measures. Even though this paper focuses on DALYs, most of the uncertainties identified play a similar role for these alternative indicators.

### Typology of uncertainty

Uncertainties in assessments about a complex world can take many forms. A typology of uncertainty can help to structure the different types of uncertainties. This can in turn help to identify useful methods and techniques to deal with the uncertainties, ranging from stakeholder discussion to sensitivity analysis.

We have adapted existing uncertainty typologies [27-33] to fit our purpose of identifying, further characterizing and dealing with the uncertainties that arise in eBoD assessments. Our typology (Table 1) distinguishes between location, nature, range, recognized ignorance, methodological unreliability and value diversity among analysts, as six characteristics of uncertainty. These characteristics apply simultaneously to a piece of uncertain information. This typology differs from the one presented by Petersen [30] in the way in which the location of uncertainty and ontic uncertainty are specified. These changes have been made in order to make the typology more applicable to burden of disease assessments. How the typology presented by Petersen differs from previous typologies such as [27,28,32] is described in [30]. It is not claimed that our typology is the best typology (cf [30]); for other purposes, other typologies might be more useful.

**Table 1:** Typology of uncertainty

Uncertainty characterizations	*Categories*	
**Location**: the location at which the uncertainty manifests itself in the assessment	***Model structure***: Structure and form of the relationships between the variables that describe the system	
	
	***Parameters***: Constants in functions that define the relationships between variables (such as relative risks or severity weights)	
	
	***Input data***: Input data sets (such as concentrations, demographic data, and incidence data)	

**Nature**: the underlying cause of the uncertainty	***Epistemic***: resulting from incomplete knowledge	
	
	***Ontic***	***Process variability***: resulting from natural and social variability in the system
		
		***Normative uncertainty***: resulting from a plurality of socio-ethico-normative considerations within a society

**Range**: expression of the uncertainty	***Statistical (range + chance)***: specified probabilities and specified outcomes	
	
	***Scenario (range + "what if")***: specified outcomes, but unspecified probabilities	

**Recognized ignorance**: unknown outcomes, unknown probabilities – uncertainties are present, but no useful estimate can be given		

**Methodological unreliability**: Methodological quality of all different elements of the assessment; a qualitative judgment of the assessment process which can based on e.g. its theoretical foundation, empirical basis, reproducibility and acceptance within the peer community		

**Value diversity among analysts**: Potential value-ladenness of assumptions which inevitably involve – to some degree – arbitrary judgments by the analysts.		

First, the location of uncertainty indicates where the uncertainty manifests itself among the main elements of the assessment. Distinction is made here between the context, model structure, parameters and input data. These locations will be further described below.

Second, the nature of uncertainty expresses whether uncertainty is primarily a consequence of the incompleteness and fallibility of knowledge, epistemic uncertainty, or primarily due to intrinsic properties of the system under study, ontic uncertainty – ontic meaning pertaining to the object. In other contexts and disciplines, ontic uncertainty is often referred to as variability. The present study distinguishes between two types of ontic uncertainty: process variability and normative uncertainty. Process uncertainty relates to variability in natural or social processes, such as the inherent variability of the weather. Normative uncertainty relates to the existence of a fundamental plurality of social, ethical or normative considerations. An example of the latter is that individuals have fundamentally different views on wellbeing and the severity of illnesses.

Third, the range of uncertainty relates to the way uncertainty can be expressed, either as a statistical uncertainty or as a scenario uncertainty. A statistical uncertainty range is appropriate when uncertainties can be adequately expressed in statistical terms, for example, as a central estimate and an interval around it. However, deeper forms of uncertainty are frequently at play. These can often not be adequately described in terms of chances or probabilities, but can only be specified in terms of a range of possible events (scenarios). In absence of information on the relative likelihood of each scenario, they are usually treated as being equally plausible. Scenario uncertainties are often construed in terms of what-if statements.

Fourth, recognized ignorance concerns those aspects of uncertainty for which we cannot establish any useful estimate, for example due to processes that have been identified but that are yet poorly understood. Unrecognized ignorance is excluded from the typology, because it concerns pure ignorance about which we cannot say anything knowledgeable: we do not know what we do not know. However, experts may acknowledge that they are ignorant about particular sources of uncertainty and that this limits the reliability of the conclusions of their studies.

Fifth, the methodological unreliability of an element of an assessment reflects weaknesses in methodological quality. It is often not possible to quantitatively establish the accuracy of a model. In those cases, one may instead use qualitative judgments to express in what ways scientific knowledge is limited. Scientific peers may judge the methodological rigor of the procedures followed. This methodological rigor can, for instance, be determined by looking at the theoretical and empirical basis, the reproducibility of the assessment and its acceptance in the peer community.

Sixth, there can be value diversity among analysts in scientific practice. Value here refers to personal values and normative judgments, instead of to numerical values. Value diversity is often reflected in the existence of alternative assumptions in the assessment. Also, assumptions made by one expert can be contested by another expert. Assessors often have considerable freedom in making choices about the design of their assessment and the interpretation of data. These choices may be influenced by different underlying epistemic, socio-cultural and practical values held by the assessors. An example of a socio-cultural value is to base the assessment on worst-case assumptions, reflecting a risk-avoiding attitude. Experts with a risk-seeking attitude may find worst-case scenarios less relevant and might prefer best-case assumptions to inform a decision [34].

The different categories of the location of uncertainty are used to structure this paper. The other characteristics are discussed in each of the sections. The various types of uncertainty will be illustrated using examples from the eBoD literature. In Table 2, some of these illustrations have been characterized according to the uncertainty typology. These examples are referred to with (Table 2, section #) in the remainder of this manuscript, in which 'section #' refers to the corresponding number in Table 2. We will also point towards methods that can be used to deal with different types of uncertainties.

**Table 2:** Illustrations of characterizations of uncertainties in environmental burden of disease assessments

Source of uncertainty		*Nature* **E**pistemic/Ontic (**Pro**cess Variability/**Nor**mative Uncertainty)	*Range* **St**atistical/**Sc**enario	*Recognized ignorance*	*Methodological unreliability*	*Value diversity among analysts*
CONTEXTUAL UNCERTAINTY						

1	Multiple ways of defining the 'total environment'	E/Nor	Sc	-	+	++

2	Only including diseases that cause at least 1% of the global burden of disease	Nor	Sc	--	--	+

MODEL STRUCTURE UNCERTAINTY						

3	Specific form of the exposure-response relationship is unknown	E	Sc	+	+	+

4	Evidence for causality (environmental factor leading to health effect) is weak and contradicting	E	Sc	++	++	+

5	Incomplete understanding of the joint effect of smoking and radon in relation to lung cancer	E	Sc	+	+	+

6	Accounting for susceptible groups if the available relative risk is not representative for this group	Pro/E	St	+	+	+

PARAMETER UNCERTAINTY						

7	Determining a relative risk (RR) for long-term exposure to PM_10_	E	St	+	+	-

8	Applying an American RR for PM_10 _to the Netherlands	E	Sc	++	+	+

9	Use of severity weights	Nor/E	Sc	+	+	++

INPUT DATA UNCERTAINTY						

10	Extrapolating non-assessment-specific exposure measurements	E	Sc	++	+	+

11	Measuring population exposure	E	St	+	+	-

### Contextual uncertainty

Contextual uncertainty stems from choices made about system boundaries and definitions used in an assessment. In eBoD studies, the definition of the environmental factor(s) considered, the associated health outcomes, the links between these, and the scenarios used in the study (including the study area, affected population, and time frame) have to be agreed upon.

#### Examples of contextual uncertainty in environmental burden of disease assessments

##### Defining environment

Defining 'environment' is not always straightforward. Whereas assessments on single risk factors can generally define exposure relatively easy, broader multiple factor analyses (about for example transport, agriculture or the total environment) need to define these boundaries more carefully.

'Environment' has been defined to exclude genetics, diet and smoking behavior, but include for instance effects of the natural environment such as dust exposure and natural disasters [2]; include physical, chemical and biological human-made or influenced exposures, but exclude occupational health and safety, the majority of traffic, war, and life-style factors [3]; include all the physical, chemical and biological factors external to the human host and all related behaviors, but exclude those natural environments that cannot reasonably be modified [5,24,35], etc. These definitions can have a significant influence on the outcome of an assessment. In technical terms, this uncertainty can be addressed by thoroughly defining the terms and scope of an assessment. However, this does not change the fact that different scopes and definitions are theoretically possible. Therefore, this contextual uncertainty (Table 2, section 1), has an epistemic component, because we can not yet gauge the complete extent of the environment; and a normative component, because different researchers hold different normative views on what the environment consists of.

##### Defining health

Many assessments define health quite clinically, including only adverse health effects that have a medical diagnosis. However, a broader definition – such as used by WHO [6] stating that health is a state of complete physical, mental and social well-being and not merely the absence of disease or infirmity – also includes less severe health effects. For instance, the burden of disease related to noise varies significantly depending on whether noise annoyance and sleep disturbance are considered health effects.

A pragmatic and normative approach to define which diseases to include in an assessment has been employed by Smith et al. [2]. They included only disease categories that cause at least 1% of the global burden of disease (Table 2, section 2). Other cut-off percentages could also have been adopted, making this source of uncertainty a form of scenario uncertainty with a normative nature and a degree of value diversity among analysts. Since it is known which diseases are excluded and why, recognized ignorance and methodological unreliability do not play a substantial role.

#### Dealing with contextual uncertainty

Results of an assessment can be very sensitive to the definitions and system boundaries chosen. Most of these definitions cannot be harmonized across assessments, because they are dependent upon the purpose of a specific assessment [36]. There is not one single way to deal with contextual uncertainties, but a few general guidelines can be given. In summary, the chosen definitions and boundaries need to be discussed, reported and consistently used [2,37]. This process may often need to involve relevant stakeholders. Even though stakeholder discussions may not reduce the uncertainties, they at least help to reveal them [1]. If more than one sensible definition can be made about an element in the assessment, thus leaving room for value diversity, multiple analyses can be run using different sets of definitions. This is especially useful when there is controversy about which definitions are most appropriate, or when the differences between definitions are considerable. Sensitivity and decision analyses can help to identify which sources of uncertainty mostly affect the final results [8-10].

### Model structure uncertainty

Model structure uncertainty relates to uncertainty about the causal structure of the modeled system: uncertainty within the boundaries chosen. Various interpretations might prevail about the dominant variables and their causal relationships. Because of the many difficulties in studying the large scale low exposure environmental health risks that are so typical of the modern Western world, different views about the model structure often exist.

#### Examples of model structure uncertainty in environmental burden of disease assessments

Even when the assessment context is agreed upon, that does not automatically mean that all potentially relevant variables are included in the assessment. For example, climate change may affect health in ways that are as yet unexpected and which can therefore not be assessed [38]. Model structure uncertainty can also relate to the applicability and form of exposure-response relationships (for instance, threshold versus no threshold, or linear versus nonlinear) (Table 2, section 3) [39,40]. Additionally, evidence for causality may not always be available or in agreement (Table 2, section 4). An example is the inconclusiveness of the evidence for an association between noise exposure and cardiovascular impacts. Some reviewers find the evidence for this relationship sufficient, whereas others state that it is limited [41]. Similarly, a WHO eBoD study [42] based their estimate of the asthma-related burden of disease for children on a relative risk that Smith [43] considered not sufficiently robust. A comparable debate runs for the long-term health impacts of air pollution, which are as yet rather uncertain. Since different decisions can be made about whether sufficient evidence for causality exists [44,45], such uncertainty is characterized as scenario uncertainty. The recognized ignorance about the existence of causality, which is partly due to methodological unreliability, could be reduced by further research, which indicates its epistemic nature.

Other examples of model structure uncertainty relate to partly unknown patho-physiological mechanisms, the use of proxies, potential latency times, vulnerable groups, co-morbidity and multi-causality. Multiple risk factors can simultaneously affect multiple health outcomes. Environmental stressors can cause health effects through intermediate factors and feedback systems. There may also be other correlated risk factors with common social and behavioral determinants [7,46-48]. When assessing such interacting risk factors, it is necessary to know whether their effects are additive (separate effects added), synergistic (separate effects multiplied), or antagonistic (separate effects reduced) [49]. Incomplete understanding of the joint effect of smoking and radon with regard to lung cancer remains a key uncertainty in assessing the risk of indoor radon [50] (Table 2, section 5). Similarly, different methods exist to account for specific susceptible population sub-groups when no representative exposure-response functions exist (Table 2, section 6).

#### Dealing with model structure uncertainty

Model structure uncertainty is often predominantly epistemic – relating to incomplete or contradictory knowledge – and more research can increase understanding and possibly reduce uncertainty.

Refsgaard et al. [51] reviewed strategies for assessing model structure uncertainty and present a framework for assessing the uncertainties of predictive models. It involves the use of multiple conceptual models, assessment of their pedigree, and reflection on the extent to which the sampled models adequately represent the space of plausible models. Additionally, sensitivity and decision analyses can provide information about the relative importance of variation between different alternative assumptions [8-10]. Widely used are also Bayesian belief networks, which can be used to assess multiple model structures [52,53].

However, resources often limit the possibility of running extensive alternative calculations, and pragmatic choices need to be made. Therefore it is most important to document the assumed conceptual and technical model structure in a transparent way, to explore and document which limitations or other viewpoints exist, and to reflect on what this means for the robustness of the results. A graphical representation of the model showing which variables and linkages are included and excluded in the assessment increases the understanding of the model structure [54]. Standardization of the way such causal diagrams are presented – all using the same convention for what certain shapes of boxes and types of arrows precisely mean – is to be recommended. Finally, if very large disagreement or ambiguity about the model structure exists, one might also consider not to carry out an eBoD study in the first place.

### Parameter uncertainty

Parameters are used to describe a relationship between variables. They can be descriptive (such as relative risks, duration estimates, or attributable fractions) or normative (such as maximum life expectancy, severity weights, policy norms, age weights and discount factors).

#### Examples of parameter uncertainty in environmental burden of disease assessments

##### Relative risks and attributable fractions

The most common descriptive parameter used in eBoD calculations is the relative risk (RR), which indicates the ratio of the risk of a disease or death among those exposed to the specified factor to those not exposed. The RR is usually derived from an epidemiological study or a meta-analysis of such studies, and subsequently applied to the specific study context. The epidemiological studies from which the RR stems can in themselves form a source of uncertainty (Table 2, section 7). The methods used to derive RRs are fairly common, which limits value diversity, at least among epidemiologists. However, uncertainty can relate to differences in study design or measurement errors, giving rise to potential methodological unreliability [1]. For some environmental risks, especially new and emerging risks such as electromagnetic fields or genetically modified foods, RR estimates are available only to a limited degree or not at all. Depending on the assessment context, it can be debated whether specific RRs can be extrapolated to other regions, time periods, substance mixtures, or population sub-groups [7,39,55]. An example is the use of RRs for the long-term effects of PM_10_, which are currently mainly available from studies in the United States. The validity of the use of such risk measures in burden of disease studies in other countries is disputable, since air pollution mixtures (for which PM_10 _is an indicator) and average population susceptibility may vary between countries [11,56] (Table 2, section 8). Similarly, in assessing the health impacts of climate change, problems have been encountered when long-term effects have had to be extrapolated from short-term associations [38].

##### Severity weights, age weights and discounting

Normative parameters such as severity weights for diseases or age weights and discounting factors for future health gains are generally based on the judgments of clinicians and economists respectively. They are therefore subjective interpretations of a number for which no 'true value' exists (Table 2, section 9) [57]. Different values will prevail depending on who is being asked, their age, gender, occupation, socio-economic status, cultural background and education level, amongst other things. This raises the question of the transferability of these weights to other situations. Estimates of severity weights also depend on the way health effects are presented to the people who are asked to make the valuation, the range of health effects to be valued in the same session, and the valuation methods [58]. As an extreme example, the severity weight for severe noise-related sleep disturbance has been estimated at 0.01 (as used in [20,59]) up to as high as 0.12 [59]. Alternative but still realistic assumptions for all normative parameters mentioned above (severity weights, age weights and discounting) can lead to major differences in DALYs, by up to a factor of four [57]. This large variation should be a reminder of the need for caution in the use of such indicators for policy purposes. If the choice of policy is sensitive to the precise value of the indicator, then an indicator with a large concealed uncertainty may be worse than none at all.

#### Dealing with parameter uncertainty

Parameter uncertainty (together with input data uncertainty, discussed next) is commonly quantitatively assessed in eBoD studies, through the use of statistical analyses. Confidence intervals (CI) of parameters such as the RR are used to calculate overall CIs for DALYs. This approach is only suitable for statistical uncertainty. However, scenario uncertainty (Table 2, sections 8 and 10) on issues for which various interpretations exist (i.e. value diversity among analysts) is more difficult to represent in CIs. For these uncertainties, similar approaches as described for contextual and model structure uncertainty can be useful: sensitivity and decision analyses in combination with transparent reporting.

### Input data uncertainty

Uncertainty in input data may relate to a range of factors, including a lack of data, inaccurate measurements, or extrapolated data. Exposure data and disease data (incidence, prevalence or mortality data) are the most common input data sets needed for eBoD calculations.

#### Examples of input data uncertainty in environmental burden of disease assessments

The greatest source of uncertainty pertaining to input data in eBoD assessments generally relates to a lack of assessment-specific measurements. For exposure data, one frequently has to rely on proxies for exposure, such as modeled environmental concentrations. Furthermore, for many risk factors, data on exposure or concentration distributions are available for only a limited number of years, regions, countries or demographic groups [5]. If no further monitoring can take place, assessors might need to extrapolate non-assessment specific data (Table 2, section 10). In climate change research, for example, impacts often relate to future exposures which cannot be measured and hence need to be modeled [38]. In a WHO study on solid fuel use across 181 countries [5,60], a combination of survey data and modeled data were used. Whether the modeled data can be meaningfully used may be judged differently by various scientists, leading to potential value diversity among analysts. Overall, Prüss-Üstün et al. [5] concluded that only for three environmental risk factors – water sanitation and hygiene, solid fuel use, and outdoor air pollution – were the necessary methodology and enough exposure data available to make sensible global estimates at country level.

Even when exposure can be measured, different measuring methods may lead to different results (Table 2, section 11). An example is the measurement of noise exposure levels, which can differ by up to 10 dB(A) depending on the methodologies used [61]. In studies related to UV radiation and skin cancer, sun exposure of many years before is often estimated using recalled sunburns or time spent in the sun. Such exposure estimates based on self-reporting can differ significantly from measurement data of ambient UV radiation levels [40].

Similar issues play a role for background morbidity and mortality data, which are needed to calculate the estimated number of attributable cases. Such data should ideally stem from empirical research or adequate monitoring in the target population [62]. However, these data are often only available at highly aggregated levels. More specific data, for example on a local scale, suffer from the small-number problem, in that estimates for rare outcomes may be highly unstable. Research shows that indicative uncertainty ranges for regional prevalence rates of 16 important diseases may range from +/- 10 percent to +/- 90 percent [39]. The common solution – modeling missing data, or extrapolating data of one country to another country – yields epistemic uncertainty.

#### Dealing with input data uncertainty

For morbidity data, models can be employed to calculate missing data and check for consistency in existing datasets. However, past trends in incidence and data inaccuracies can lead to large discrepancies between measurements and model calculations, and their use requires both caution and expert knowledge [47,62,63].

A data quality assessment can be used to evaluate whether input data are suitable for the intended purpose. Such an assessment involves "the scientific and statistical evaluation of data to determine whether they meet the objectives of the project, and thus are of the right type, quality, and quantity to support their intended use" [64]. The Numerical, Unit, Spread, Assessment and Pedigree (NUSAP) system [27,65,66] is another method to assess data quality. In addition to the more standard quantitative uncertainty assessment (number, units and the spread of those numbers), the NUSAP approach also includes an evaluation of the reliability of the information (assessment) and its the scientific basis (pedigree).

### Using the uncertainty typology in practice

In the preceding paragraphs we have outlined ways to deal with various types of uncertainties. In practice, eBoD assessments do not only have to deal with uncertainties, but also with time and budget constraints. It might often not be possible to employ all possible methods to deal with all the uncertainties inherent in the assessment. Therefore, it is necessary to prioritize uncertainties and the work needed to assess or reduce them. Here we shortly describe how to 1. identify and characterize sources of uncertainty; 2. prioritize sources of uncertainties; and 3. select and apply methods for dealing with uncertainties. We will describe how, in all these steps, the uncertainty typology can be used to support the process. Subsequent communication of the results to policy makers will be discussed in the following paragraph.

1. First, the different sources of uncertainty are to be identified. The generation of this longlist of uncertainty sources can be done using two different approaches: 1) by analyzing each step of the eBoD assessment at hand and subsequently characterizing each source according to the typology, and 2) by considering each possible type from the uncertainty typology and discussing where in the assessment this type of uncertainty may occur. Reasoning from both angles may help to minimize the chance that uncertainty sources are overlooked. The resulting list of uncertainties can be further characterized using the uncertainty typology.

2. The relative importance of each uncertain element can subsequently be weighted, based on its potential impact on the outcome of the eBoD assessment in question. Where some form of quantification is possible, the relative importance can be assessed by means of sensitivity analysis [8-10]. However, for many sources of uncertainty, such quantification is not feasible. In that case, the relative importance can be assessed using expert judgment. Two possible approaches include coding and card sorting. In the coding approach [67], experts are asked to go over the longlist of uncertainty sources and code each source as being either of a) a crucial importance; b) an average importance; c) a medium importance or d) a low importance. This is a quick and dirty technique and, to avoid errors and biases, several experts should do this independently and discuss potential differences in their judgments. The card sorting approach (used by e.g [68]) is more advanced and involves organizing an expert workshop. Experts are asked to independently select the top 20% (or another percentage) sources of uncertainty that they consider most important in view of their impact on the eBoD calculation at hand, and sort these according to importance. The uncertainties are displayed on cards to facilitate the sorting – hence the name. Results from individual experts are combined to arrive at a group ranking of the items on the longlist. Arguments used by the experts to defend their ranking need to be documented and special attention should be given to reasons for any substantial disagreement on the importance of a particular uncertainty source.

3. Once the prioritization has been done, suitable tools can be selected for further analysis of the key uncertainties identified. Each uncertainty type may require a different method to address it, and to gauge its impact on decision making. The uncertainty tool catalogue by Van der Sluijs et al. [69] provides guidance for selecting appropriate methods that match the characterization of the uncertainty in the typology. Refsgaard et al. [70] also describe various methods for dealing with uncertainties, and explain which purposes they may serve.

It may not be possible to correctly identify, characterize and prioritize all sources of uncertainty in the beginning of an assessment. The typology may thus need to be reassessed throughout the project. New sources of uncertainty may be added or their weights may be adjusted. The uncertainty typology should therefore be used interactively throughout the study. As such, it also provides a framework to keep track of all sources of uncertainty, so that sources identified early in the project – especially those that cannot be quantified – are not forgotten at the end of the study, when results are reported.

### Communicating uncertain results to policy makers

Most policy makers will feel more comfortable when making decisions based on single, undisputed numbers with small uncertainty ranges, than on ambiguous or controversial estimates and scenario analyses. However, unfortunately that is often not the way complex processes can be described. On the other hand, giving policy makers a lengthy report listing all the possible uncertainties will not necessarily lead to informed policy making either. Scientists can help policy makers by assessing which uncertainties are most relevant for the policy decisions to be made. They can identify policy options that are robust given these uncertainties. If no single best policy option for all scenarios can be determined, all reasonable options can be discussed in a democratic process including scientists, stakeholders, policy makers and politicians [71]. As the communication needs of all these parties can vary greatly, a single mode of risk communication is rarely sufficient.

Uncertainties can be communicated linguistically, numerically, or graphically. Confidence intervals can be provided reflecting uncertainty in parameters and input data. For uncertainties that cannot be expressed in statistical intervals, other characterizations of likelihood can be used. Risbey et al. [72] have proposed expressions for different levels of precision, ranging from full well defended probability density functions, to percentile bounds, first order estimates, expected signs or trends, ambiguous signs or trends and, finally, effective ignorance. Many of the uncertainties identified in our study cannot be captured quantitatively, but some can be expressed in these latter characterizations of precision. Additionally, if any policy recommendations are made, the strength of these recommendations and the quality of the underlying evidence can be expressed using a uniform grading system [73,74]. Such a systematic and explicit approach to judging the quality of evidence and the strength of recommendations can facilitate appraisal of these judgments, and improve communication [73,74]. Providing a graphical representation of the underlying model in a standardized way can support further understanding of the assessment context and model structure.

In order not to overwhelm the user of the assessment results with uncertainties, the concept of progressive disclosure of information can be employed [75,76]. This involves tailoring the information about uncertainty to the target audience. In a press release or a project summary, for example, the uncertainties that are most relevant to the final policy decisions need to be described, without any technical details. As such, a policy maker using the results of an eBoD assessment will not be directly confronted with a typology of all uncertainties, but will be provided with the information needed to properly interpret the results. The main assessment report may subsequently contain more detailed information, with emphasis on the nature, extent and sources of uncertainties. Ideally, it presents all methods, assumptions, parameters and input data, thereby providing maximum transparency of the assessment approach. Even though DALYs are made to reduce complex information to single numbers, it is essential to allow readers to unravel the DALYs and, when desired, reproduce them [11,57] or recalculate any estimates using their own data or assumptions [77].

The assertion that burden of disease figures can only be properly interpreted when presented with assessment-specific, informative and complete background information leads to a second consideration related to the usability of assessment results. They can only be used for the specific purpose for which they were derived, and should not be used in other assessments or for other policy purposes.

## Discussion

Disability Adjusted Life Years – or other forms of aggregated health measures – can be very attractive indicators for policy makers. The measure combines information about the magnitude, severity and duration of adverse health effects into one number, thereby providing a means to compare otherwise incomparable environmental health problems. This simplification of the complex underlying reality is the defining advantage of the measure, but it also presents pitfalls. We have shown that various types of uncertainty can influence environmental burden of disease (eBoD) assessments and their output, thereby potentially influencing policy decisions based on these assessments. Statistically quantifiable uncertainty in parameters and input data – the type of uncertainty that is usually well communicated in eBoD assessments – is far from the only type of uncertainty, or even the most important. Variations in definitions of the environment, the health effects, and the scenarios assessed, unknown impacts of multi-causality and co-morbidity, lacking consensus about causality, controversial views about model structures, and many other sources of uncertainty may affect eBoD assessments, but cannot be easily quantified, and are usually not fully addressed.

The use of a typology to characterize and structure uncertainties can help to deal with them. Dealing with uncertainties does not necessarily mean reducing them. Much of the time, mere identification and proper communication of uncertainties along with systematic reflection on their policy implications is most important, or – more practicably – the only feasible thing to do. Ideally, policies should be robust under the uncertainties that are identified.

The potential extent of uncertainties presented here should not be interpreted as criticism of the DALY approach as such, or as an argument for not using the method. Instead, for some assessments, DALYs can be a very valuable way of presenting the possible extent of environmental health effects to policy makers. Uncertainties do not halt eBoD assessments, but do affect the assessment process and the interpretation and communication of its results. Scientists have the responsibility to assess and communicate assessments in such a way that underlying uncertainties are reflected in the outcomes. Results should not be presented as being more robust than can be inferred from the underlying knowledge base. And policy makers, for their part, have the responsibility to take information about uncertainty seriously and deal with it sensibly [78]. If not, then the interface between science and policy needs to be re-designed, lest misdirected policies be based on a false precision of scientific inputs.

In the meantime, on a meta-level, the methodology for calculating the eBoD and dealing with uncertainties needs to be improved [58]. For example, there should be a study of the disproportionate way in which the uncertainty in small severity weights (such as severe noise-related sleep disruption [20,59]) affects overall assessment uncertainty.

The main limitation of the typology presented here is that it strongly relies on expert judgment and mainly yields qualitative insights. Its main application should be to precede and supplement quantitative uncertainty analysis, and not to replace it. In addition, further research on summary measures can perhaps in the future lead to better measures than DALYs, an area already explored by Murray [58]. It is not possible to completely harmonize methods and knowledge or to standardize datasets. These are often highly assessment-specific, and should stay that way. However, the criteria used for determining which methods and datasets to use should be harmonized as much as possible. Overall, it would be useful to further study the pitfalls of these types of aggregated indicators, and to develop methods to identify and prevent the hyper-sensitivity of policy decisions to overly precise indicators.

## Conclusion

Increased awareness of the issue of uncertainty and a well-structured approach towards assessing and communicating uncertainties can help to bring about a more balanced interpretation of the results of eBoD assessments. A typology of uncertainties such as presented in this paper can be used to systematically identify and map key uncertainties. As such, it precedes and complements quantitative uncertainty assessment. The use of a typology may facilitate a structured dialogue between scientists and stakeholders on possible sources and types of uncertainty. This may help the key actors to achieve a common understanding of the uncertainties and their importance.

## Abbreviations

CI: Confidence Interval; DALE: Disability Adjusted Life Expectancy; DALYs: Disability Adjusted Life Years; eBoD: Environmental burden of disease; HALE: Health Adjusted Life Expectancy; HALYs: Health Adjusted Life Years; PM: Particulate Matter; QALYs: Quality Adjusted Life Years; RR: Relative Risk; UV: Ultraviolet; WHO: World Health Organization

## Competing interests

The authors declare that they have no competing interests.

## Authors' contributions

AK initiated the research and studied the environmental burden of disease literature. AP and JPS designed and described the uncertainty typology. All authors discussed the application of the uncertainty typology to the case of environmental burden of disease assessment. AK drafted the main manuscript using input from ACP, JS and EL. All authors read and approved the final manuscript.
